# Polyphenol-Based Nanoparticles: A Promising Frontier for Enhanced Colorectal Cancer Treatment

**DOI:** 10.3390/cancers15153826

**Published:** 2023-07-27

**Authors:** Hicham Wahnou, Bertrand Liagre, Vincent Sol, Hicham El Attar, Rukset Attar, Mounia Oudghiri, Raphaël Emmanuel Duval, Youness Limami

**Affiliations:** 1 Laboratory of Immunology and Biodiversity, Faculty of Sciences Ain Chock, Hassan II University, B.P. 2693, Maarif, Casablanca 20100, Morocco; hwwahnou@gmail.com (H.W.); mounia.oudghiri@univh2c.ma (M.O.); 2 Univ. Limoges, LABCiS, UR 22722, F-87000 Limoges, France; bertrand.liagre@unilim.fr (B.L.); vincent.sol@unilim.fr (V.S.); 3 Annasr Pathology Center, El Jadida 24000, Morocco; cpa86513@gmail.com; 4 Department of Obstetrics and Gynecology, Yeditepe University, Istanbul 34280, Turkey; ruksetattar@hotmail.com; 5 Université de Lorraine, CNRS, L2CM, F-54000 Nancy, France; 6 Laboratory of Health Sciences and Technologies, Higher Institute of Health Sciences, Hassan First University of Settat, Settat 26000, Morocco

**Keywords:** colorectal cancer, nanomedicine, nanoparticles, polyphenols, natural compounds, chemical synthesis, drug delivery system, signaling pathways, cancer cell death

## Abstract

**Simple Summary:**

Conventional therapies for the treatment of colorectal cancer induce several side effects that impact the effectiveness of current therapies as well as the quality of patients’ life. In recent years, natural compounds with anticancer properties have gained attention as potential therapeutic agents for various cancers including colorectal cancer. However, several natural compounds such as polyphenols are facing obstacles for their use as anticancer drugs, such as intrinsic poor solubility, plasmatic instability, ineffective cellular uptake, and biological barriers. Currently, novel approaches in precision medicine and nanomedicine are being developed. In this context, to harness the full potential of natural compounds, researchers have explored the use of nanoparticles as a drug delivery system for targeted and enhanced therapeutic efficacy as well as limited side effects. This review provides data on recent advances in strategies using polyphenols-based nanoparticles for the treatment of colorectal cancer.

**Abstract:**

Colorectal cancer (CRC) poses a significant challenge in healthcare, necessitating the exploration of novel therapeutic strategies. Natural compounds such as polyphenols with inherent anticancer properties have gained attention as potential therapeutic agents. This review highlights the need for novel therapeutic approaches in CRC, followed by a discussion on the synthesis of polyphenols-based nanoparticles. Various synthesis techniques, including dynamic covalent bonding, non-covalent bonding, polymerization, chemical conjugation, reduction, and metal-polyphenol networks, are explored. The mechanisms of action of these nanoparticles, encompassing passive and active targeting mechanisms, are also discussed. The review further examines the intrinsic anticancer activity of polyphenols and their enhancement through nano-based delivery systems. This section explores the natural anticancer properties of polyphenols and investigates different nano-based delivery systems, such as micelles, nanogels, liposomes, nanoemulsions, gold nanoparticles, mesoporous silica nanoparticles, and metal–organic frameworks. The review concludes by emphasizing the potential of nanoparticle-based strategies utilizing polyphenols for CRC treatment and highlights the need for future research to optimize their efficacy and safety. Overall, this review provides valuable insights into the synthesis, mechanisms of action, intrinsic anticancer activity, and enhancement of polyphenols-based nanoparticles for CRC treatment.

## 1. Introduction

**Figure 1:**
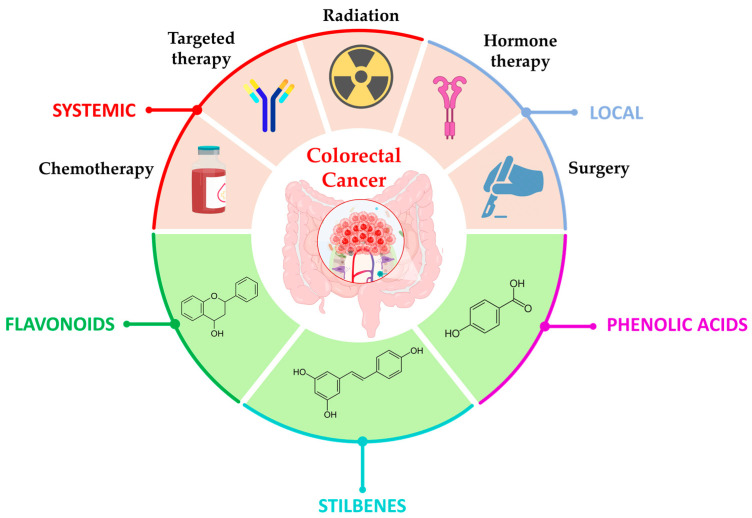
Therapeutic approaches for CRC, with conventional treatment (i.e., surgery, radiation, hormone therapy, targeted therapy, and chemotherapy) and some types of polyphenols used in CRC treatment (i.e., flavonoids, stilbenes, and phenolic acids).

Colorectal cancer (CRC) remains a significant healthcare challenge, necessitating the exploration of novel therapeutic strategies. It accounts for over 1.1 million new cases and approximately 500,000 deaths worldwide each year, making it one of the most common malignant tumors with severe implications for public health [1,2]. Traditional treatment approaches for cancer, including surgery, chemotherapy, hormone therapy, and targeted therapies (Figure 1), have limitations such as drug resistance, systemic toxicity, and a lack of selectivity towards cancer cells [3,4,5]. As a result, there is a growing interest in the development of alternative treatment options that can overcome these challenges and provide more effective outcomes [6,7,8,9,10,11,12].

In recent years, natural compounds with inherent anticancer properties have gained attention as potential therapeutic agents for various cancers, including CRC [13]. Among these compounds, polyphenols have emerged as a promising class of bioactive molecules. These compounds, derived from diverse sources such as plants and marine organisms, exhibit multiple bioactive properties that can suppress tumor growth, induce apoptosis, inhibit angiogenesis, and modulate cellular signaling pathways involved in cancer progression [14,15,16,17,18,19,20,21,22,23,24,25,26]. To harness the full potential of natural compounds such as polyphenols, researchers have explored the use of nanoparticles (NPs) as a delivery platform for targeted and enhanced therapeutic efficacy [27,28,29,30]. NPs derived from natural compounds offer several advantages, including improved stability, solubility in biological media, controlled release of the encapsulated compounds, enhanced cellular uptake, and the ability to overcome biological barriers [30]. In the case of CRC, these NPs can be administered through oral and rectal routes, offering non-invasive and patient-friendly delivery methods [31,32]. Oral administration allows for direct exposure of polyphenols to the colon tissues, taking advantage of the colon’s absorption mechanisms, prolonged transit time, and presence of microflora. This approach enhances the bioavailability of polyphenols and their targeted delivery to the colon, potentially improving treatment outcomes [31]. Similarly, the rectal route provides an option that allows for localized delivery of NP-based therapies to the colorectal tumor site. It bypasses first-pass metabolism and reduces systemic toxicity, minimizing exposure to healthy tissues and potential side effects [32]. Furthermore, the rectal route offers an alternative for patients with swallowing difficulties or gastrointestinal complications, enhancing treatment accessibility and patient compliance. By combining the properties of polyphenols, the unique characteristics of the colon, and the benefits of oral and rectal administration, NPs-based therapies present an effective and less toxic approach for targeting CRC. Moreover, the unique physicochemical properties of NPs can be tailored to optimize their performance as drug carriers, improving the therapeutic outcomes [33].

This review aims to provide a comprehensive overview of the application of NPs derived from polyphenols as potential treatments for CRC. The focus will be on discussing the synthesis methods, physicochemical properties, and mechanisms of action of polyphenols-based NPs against CRC cells. Additionally, we will explore the potential of various polyphenols-based NPs that have demonstrated promising anticancer properties. The review will also address preclinical studies conducted in this field and highlight the challenges associated with utilizing polyphenols-based NPs for effective CRC treatment.

## 2. Polyphenols-Based NPs: Synthesis and Mechanisms of Action

### 2.1. Synthesis

#### 2.1.1. Dynamic Covalent Bonding

**Figure 2:**
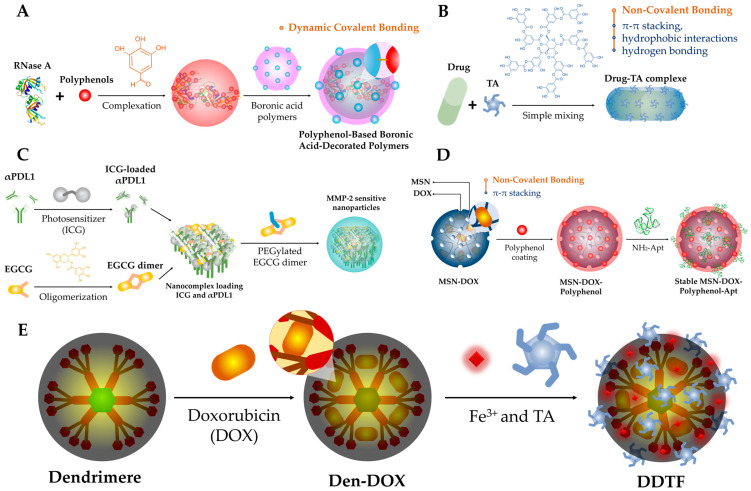
Graphically simplified representation of the different approaches for Polyphenol-based NPs synthesis. (**A**) Intracellular protein delivery enhanced by polyphenol-based boronic acid-decorated polymers: complexation of RNase A and polyphenols by Schiff’s base reactions followed by a second complexation to boronic acid polymers thanks to reaction between boronic acids and diol-based molecules [30]; (**B**) non-covalent bonding of hydrophobic drugs and tannic acid (TA); (**C**) fabrication of MMP-2-sensitive S-αPDL1/ICG nanoparticles: complexation of αPDL1 with photosensitizer ICG, stabilization with dEGCG, and compression by PEGylated EGCG dimer [46]; (**D**) polyphenol-coated mesoporous silica nanoparticles (MSNs) for cancer therapy: GSH-responsive polyphenol-coated MSNs loaded with doxorubicin (DOX) were developed for targeted cancer treatment: the preparation was based on EGCG-modified mesoporous silica nanoparticles (MSNs) for drug delivery purposes. The anticancer drug DOX was loaded into the MSNs through noncovalent adsorption, forming the MSN-DOX complex. The amine-terminated DNA aptamer made the complex physiologically stable and could be degraded under an acidic environment and by intracellular GSH, resulting in the release of drugs [30]; (**E**) fabricated Den−DOX−TA−Fe^3+^ (DDTF) nanocomplexes for delivering DOX to the nuclei via coating the DOX−Den complex with the TA−Fe^3+^ metal–polyphenol networks [47].

Polyphenols have been utilized in the fabrication of smart materials for biomedical applications by forming polymers through dynamic covalent bonds [34]. These dynamic bonds can break and reform in response to stimuli such as pH, temperature, and hydroxylated molecules [30]. Boronic esters, formed through the reaction between boronic acids and diol-based molecules, have been used to create polyphenol-based nanoassemblies with high stability and responsiveness (Figure 2A) [35]. Additionally, reversible Schiff’s base reactions, involving imine group formation, have been also employed to prepare polyphenol-based nanoassemblies [36]. These reactions enable the modification of proteins and the formation of nanocomplexes with enhanced stability and triggered drug release in response to specific environmental factors. For example, polyphenols have been conjugated to proteins, such as bovine serum albumin (BSA) and RNase A via Schiff’s base reactions, allowing for the controlled release of proteins in response to factors like lysosomal acidity and anionic molecules (Figure 2A) [27].

#### 2.1.2. Non-Covalent Bonding

Polyphenols exhibit a strong affinity for various molecules, including DNAs, proteins, peptides, and polymers, through non-covalent interactions. These interactions involve electrostatic interactions, hydrophobic interactions, hydrogen bonding, and π-π stacking [30]. For example, polyphenols such as tannic acid have been found to interact with proteins through multiple non-covalent interactions, such as electrostatic interactions, hydrogen bonding, π-π stacking, hydrophobic interactions, and van der Waals forces [30,37,38]. Moreover, polyphenols also bind to DNA and RNA through π-π stacking, hydrophobic interactions, and hydrogen bonding [39,40]. This binding ability has been utilized in the development of functional materials, such as microcapsules, multilayer films, and NPs, by combining polyphenols with natural or synthetic polymers. Furthermore, polyphenols can interact with small molecules, including hydrophobic drugs (e.g., Herceptin), through π-π stacking, hydrophobic interactions, and hydrogen bonding (Figure 2B) [41]. These interactions have been harnessed to create stable drug delivery systems with high drug loading capacities.

#### 2.1.3. Polymerization

Polymerization of polyphenols is a natural phenomenon that occurs in various scenarios. In red wine aging, exposure to low levels of oxygen promotes the polymerization of tannin molecules, resulting in a smoother taste [42]. Similarly, the precursor dopamine can self-polymerize in mildly alkaline solutions to form polydopamine (PDA), a major pigment in natural melanin [43]. Inspired by these processes, researchers have synthesized polymeric condensed polyphenols, such as oligomerized epigallocatechin-3-O-gallate and oligomeric catechins, through intermolecular polycondensation reactions. These polyphenol polymers can assemble into nanostructures by interacting with biological molecules or undergoing oxidative coupling with the help of metal ions as catalysts [44]. The resulting oligo/polymeric polyphenol nanoassemblies exhibit aggregation and assembly driven by noncovalent interactions (Figure 2C). These nanoassemblies can have controlled size and surface properties, and they maintain the characteristic features of polyphenols after polymerization [30,45]. They can also be disassembled by reducing agents and are resistant to concentrated hydrochloric acid but decompose under weakly alkaline conditions [45]. The exact mechanism of coating formation is still not fully understood but is believed to involve oxidation followed by oligomerization and surface deposition driven by the affinity of polyphenols for surfaces [30].

#### 2.1.4. Chemical Conjugation

Polymer-based nanoplatforms with self-assembling properties have been extensively studied for biomedical applications, particularly for drug delivery [48,49]. These platforms utilize amphiphilic polymers that can spontaneously form nanomicelles with defined core–shell structures in suitable solvents [48]. Researchers have focused on developing stable micellar nanocomplexes (MNCs) by chemically conjugating polyphenols with polymers. These MNCs consist of a water-insoluble core for drug encapsulation and a water-soluble shell, often made of polyethylene glycol (PEG), to provide stealth properties during circulation. Examples include PEG-conjugated epigallocatechin-3-O-gallate (PEG-EGCG) and PEG-EGCG in combination with anticancer drugs, which self-assemble into MNCs with high drug-loading capacity and stability (Figure 2D) [41]. Hyaluronic acid (HA) has also been used for micelle fabrication, with EGCG linked to HA to enhance interactions and targeting capabilities [50,51]. The incorporation of polyphenols into MNCs allows for improved stability, solubility, and reduced cytotoxicity of hydrophobic drugs [52]. These polyphenols-based MNCs hold promise for advanced drug delivery in biomedical applications.

#### 2.1.5. Reduction

The development of environmentally friendly methods for preparing NPs has gained attention due to concerns about their biological and environmental impacts (Figure 2E). Polyphenols, such as tea polyphenols, have emerged as promising reducing agents for the green synthesis of metal NPs [53]. They possess the ability to donate hydrogen atoms or electrons, making them effective in reducing and capping metal NPs [54]. Tea polyphenols have been successfully used to prepare silver and gold (Au) NPs, resulting in well-dispersed NPs with controllable sizes and shapes [54,55]. These methods are advantageous compared to physical and chemical strategies that involve high pressure, temperature, and toxic substances. Furthermore, the reducing capacity of polyphenols extends to the fabrication of inorganic nonmetallic materials, including graphene-based materials and carbon nanotubes [56,57]. Polyphenols, such as EGCG, have been utilized to disperse single-walled carbon nanotubes and reduce graphene oxide to functional graphene nanosheets [56]. These eco-friendly approaches offer possibilities to produce biocompatible graphene for various biomedical applications.

#### 2.1.6. Metal–Polyphenol Networks (MPNs)

The coordination ability of the catechol structure in natural polyphenols with various metal ions has led to the development of MPNs with flexible structures, pH responsiveness, and thermal stability [58]. The fabrication of MPNs can be achieved through different approaches. The one-step mixing method involves the simple mixing of metal ions and polyphenols in the presence of a substrate, resulting in the formation of an MPN coating [30,59]. This method is cost-effective and scalable, with substrates such as polystyrene, mesoporous silica nanoparticles, calcium carbonate, and metal–organic frameworks being used [59,60]. Multistep assembly is another approach where substrates are sequentially incubated with polyphenol and metal ion solutions (Figure 2E). The MPNs formed through this method have controlled growth rates based on pH, and the chelation sites of polyphenols are mostly occupied by metal ions [61]. The sol–gel method is an alternative approach where pre-crosslinked polyphenol oligomers are combined with metal ions to form MPN nanospheres [62]. This method is template-free and offers a facile synthesis route for MPNs [62].

### 2.2. Mechanisms of Action

To enhance tumor targeting in NP-based drugs, researchers have focused on understanding tumor biology and the interaction between nanocarriers and tumor cells. Targeting mechanisms can be classified into passive and active targeting.

Passive targeting exploits the differences between tumor and normal tissue to deliver drugs effectively [63]. It relies on the enhanced permeability and retention (EPR) effect, allowing macromolecules, including NPs, to accumulate in tumors [64,65]. The size of NPs affects their penetration, while the acidic tumor microenvironment caused by glycolysis triggers the release of drugs (Figure 2A,B,D) [66,67]. However, passive targeting has limitations, such as non-specific drug distribution and variations in the EPR effect across different tumors.

The second mechanism by which NPs targets tumor is active and require ligand/receptor recognition. Ligands on the surface of NPs are chosen to target molecules that are overexpressed on cancer cell surfaces, enabling differentiation between targeted cells and healthy cells [68]. This interaction triggers receptor-mediated endocytosis, facilitating the internalization and successful drug release of NPs (Figure 2C) [69]. Active targeting is particularly suitable for delivering macromolecular drugs like proteins and siRNAs [70]. Various ligands such as monoclonal antibodies, peptides, amino acids, vitamins, and carbohydrates can be used, targeting receptors including the transferrin receptor, folate receptor, glycoproteins, and the epidermal growth factor receptor (EGFR) [67,70]. These ligand-receptor interactions play a crucial role in achieving targeted drug delivery.

## 3. Polyphenol-Based NPs: Intrinsic Anticancer Activity and Enhancement

### 3.1. Polyphenols-Intrinsic Anticancer Properties

Polyphenols, a diverse class of bioactive compounds, exhibit a distinctive chemical structure characterized by multiple phenolic rings and various functional groups. They can be classified into different subclasses based on their chemical structure and origin (Figure 1) [71]. Flavonoids, which include flavones, flavonols, and flavanones, constitute one major subclass, while phenolic acids such as hydroxybenzoic acids and hydroxycinnamic acids represent another. Additionally, stilbenes, such as resveratrol and other subclasses, contribute to the broad range of polyphenols [71]. Examples of commonly studied polyphenolic compounds in the context of CRC treatment include EGCG found in green tea, curcumin from turmeric, quercetin present in fruits and vegetables, resveratrol obtained from grapes, and genistein found in soybeans. These polyphenols possess unique chemical structures that contribute to their remarkable anticancer activity (Table 1).

Polyphenolic compounds such as EGCG, quercetin, tannic acid, and resveratrol possess anticancer properties by targeting various cancer hallmarks including inhibition of proliferative signaling, induction of cell death, and modulation of gut microbiota.

Polyphenols can induce cell cycle arrest by targeting various signaling pathways. EGCG, for example, can interact and inhibit receptor tyrosine kinases (RTKs), which are cell surface receptors that play a key role in the activation of the survival PI3K/Akt signaling pathway [72]. Additionally, these compounds can modulate gene expression through epigenetic mechanisms, such as DNA methylation and histone modification, to inhibit tumor growth [73,74,75,76]. Quercetin, on the other hand, can cause S phase arrest by decreasing the protein expression of CDK2, cyclins A and B while upregulating p53 and p57 proteins [77]. Quercetin can also act as a prooxidant molecule causing DNA damage and resulting in cell cycle arrest and/or p53-dependent or independent mitochondrial apoptosis [78]. Similarly, resveratrol can inhibit Akt, STAT3 signaling pathways to block cell in dosage dependent manner [24].

Cell death can also be caused by polyphenolic compounds. EGCG has demonstrated the ability to increase the stability and transcriptional activity of the tumor suppressor p53, leading to apoptosis [20]. Furthermore, EGCG has also been described to induce autophagy through the inactivation of the PI3K/Akt/mTOR signaling pathway [72]. Similarly, quercetin triggers apoptosis in cancer cells by reducing the expression of Bcl-2 through a mitochondria-mediated pathway [21]. Additionally, quercetin treatment has been found to induce protective autophagy by modulating Akt/mTOR signaling and activating HIF-1α signaling, thereby counteracting quercetin-induced apoptotic cell death and affecting its therapeutic effectiveness [22]. Furthermore, resveratrol has also been investigated for its potential to induce cell death such as, apoptosis and autophagy in various cancers, including CRC, by modulating signaling pathways such as caspase-3, caspase-8, Poly (ADP-Ribose) Polymerase (PARP), LC3-I, LC3-II and PI3K/Akt/mTOR [24,25,26]. Necroptosis may also be induced by resveratrol by increasing the levels of p-RIPK3 and p-MLKL [26].

Finally, polyphenols have also been investigated to modulate microbiome polymorphic variability—one of the newly described hallmarks of cancer. Microbiome can influence cancer phenotypes, development, and progression, with specific effects observed in CRC [79]. In fact, the balance between cancer-protective and cancer-promoting microbiomes modulate the incidence and pathogenesis of CRC as well as response to therapy [79]. Polyphenolic compounds have been shown to modulate gut microbiota, affecting the development of CRC [15]. In a model of CRC in mice, supplementation of polyphenols such as isoliquiritigenin, anthocyanin, and EGCG altered the gut microbial composition towards a healthier profile [15]. Additionally, polyphenols can indirectly inhibit tumor growth by influencing the behavior of cells in the tumor microenvironment [14]. For instance, castalagin improves the efficacy of immune therapy by recruiting beneficial gut bacteria [80]. In addition, Musial et al. reported several lines of evidence that support anticancer effects of polyphenols from coffee and green tea extracts towards various cancers including CRC [81].

**Figure 3:**
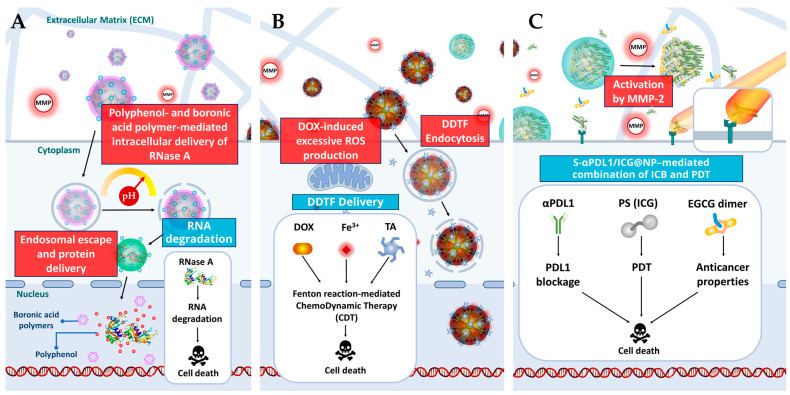
Overview of the mechanisms of action of some nano-based drug delivery of natural polyphenolic compounds. (**A**) Polyphenol-based intracellular protein delivery by boronic acid-decorated polymers. The presence of polyphenols increased the affinity between boronic acid-containing polymers and proteins. In acidic environments, the pH-responsive catechol–boronate bonds formed between the boronic acid-conjugated polymers and polyphenols allowed for the release of the RNase that can cause cell death by destroying targeted RNA. (**B**) DOX−Den complex with the TA−Fe^3+^ MPN for chemodynamic therapy (CDT). DDTF efficiently transports DOX into cancer cells by evading drug efflux transporters on the plasma membrane. Inside the cells, DOX is delivered to the nuclei through the Fenton reaction-mediated CDT. The excessive production of reactive oxygen species (ROS) induced by the Fenton reaction and DOX ultimately leads to the elimination of drug-resistant cancer cells. (**C**) MMP-2-sensitive PEGylated EGCG dimer and EGCG dimer facilitated combination immune checkpoint blockade and photodynamic therapy using an αPD-L1/ICG nanocomplex. Once the nanoparticle is activated by MMP-2, it releases αPD-L1/ICG, and the antibody blocks the PLD1 checkpoint, whereas the illumination of the photosensitizer induces various effects including ROS generation and cell death.

The efficacy of polyphenols has also been a subject of investigation in clinical trials, highlighting their potential in CRC treatment. By conducting research on clinical trials website to further explore the potential of polyphenols, two completed studies using the keywords “polyphenols” and “colorectal cancer” were found [82]. The first trial, registered as NCT01916239, examined the use of pomegranate extract supplementation in CRC patients as a potential intervention. It aimed to evaluate the impact of pomegranate extract supplementation on biomarkers associated with CRC including metabolic and gene expression profiling. The second trial, registered as NCT01360320, focused on green tea extract and its therapeutic potential. This study aimed to assess the preventive effects of green tea extract on adenomas, which are precursor lesions of CRC. These clinical trials demonstrate the growing interest in investigating the efficacy and safety of polyphenols as promising interventions for CRC treatment, providing valuable insights into their potential benefits. Despite the diverse mechanisms by which polyphenols can destroy CRC cells, the limitations of these compounds present challenges to their extensive utilization in medical research [83,84,85]. Issues such as poor chemical stability, low water solubility, limited bioavailability, rapid elimination from the system, and quick metabolism hinder their broader application [84]. However, significant progress has been made in the field of biological materials and drug delivery strategies, allowing researchers to effectively address these issues [86,87,88,89]. Encapsulation of therapeutic polyphenols within drug delivery systems has emerged as a promising approach to enhance their therapeutic effects (Figure 3).

### 3.2. Polyphenols Properties Enhancement via Nano-Based Delivery Systems

**Figure 4:**
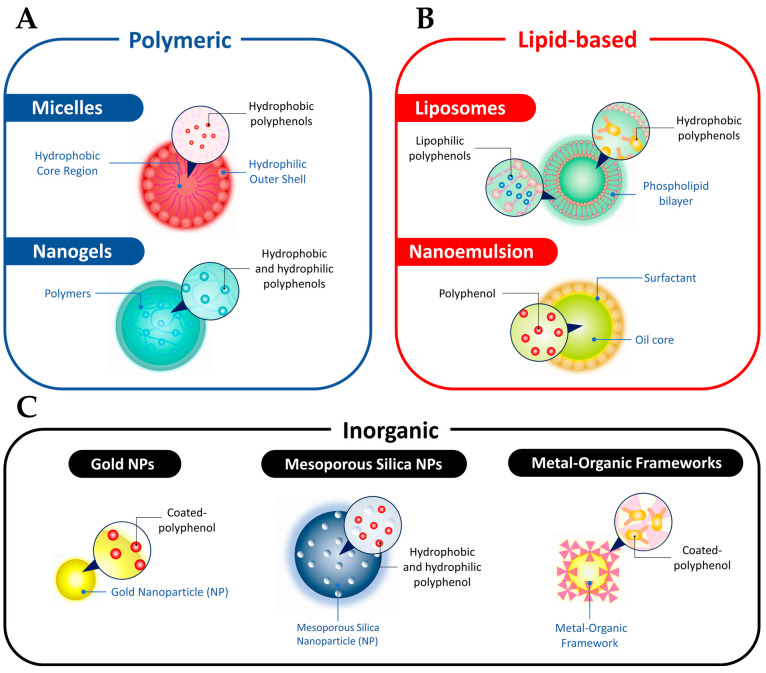
Classification of nanocarriers that are commonly utilized to deliver polyphenols compounds in cancer therapy. (**A**) Polymeric NPs, (**B**) lipid-based NPs, (**C**) inorganic NPs.

Nano-based delivery strategies enable the simultaneous administration of multiple functional drugs, enhancing the potential of polyphenolic compounds in cancer therapy. Nanocarriers that are commonly utilized to deliver natural polyphenolic compounds in cancer therapy, including in the context of CRC treatment, are micelles, nanogels, liposomes, nanoemulsions, AuNPs, MSNs, and metal–organic frameworks (MOFs) (Figure 4). In this section, we will discuss the structure and classification of nanoparticles, their role in enhancing the anticancer properties of polyphenols, and the challenges associated with NP-based delivery systems.

#### 3.2.1. Micelles

Micelles, as nanocarriers, hold great promise for targeted delivery of polyphenolic compounds in CRC therapy. These self-assembled structures consist of a hydrophilic polymeric shell and a hydrophobic core, offering advantages such as small size and enhanced permeability at lesion sites (Figure 4A) [90]. In CRC treatment, micelles can effectively deliver polyphenolic compounds like resveratrol and curcumin [91,92]. For example, nanomicelles loaded with hypoxia modulator resveratrol and photodynamic reagent chlorin-e6 have shown potential in triggering autophagic cell death and apoptosis of oral squamous cell carcinoma cells [91]. Glutathione (GSH)-sensitive nanomicelles integrated with curcumin have also been designed to target and treat esophageal cancer [92]. Additionally, micelles improve the solubility of polyphenolic compounds, as seen with the nano poly(lactic-co-glycolic acid) (PLGA)-curcumin micelle, which reverses gemcitabine resistance in CRC by suppressing the nuclear factor-κB (NF-κB) signaling pathway [91]. These findings highlight the potential of micelles as effective nanocarriers for delivering polyphenolic compounds in CRC therapy, addressing solubility issues, and enhancing treatment outcomes.

#### 3.2.2. Nanogels

In addition to micelles and liposomes, nanogels have emerged as another promising type of nanocarrier for targeted delivery of therapeutic agents in CRC therapy. With their porous structures and large, surface-to-volume ratios, nanogels can encapsulate both hydrophilic and hydrophobic therapeutic agents (Figure 4A) [93]. These nanocarriers enhance drug permeability and retention at tumor sites, improving treatment efficacy. For instance, TME-responsive nanogels loaded with resiquimod and EGCG have been developed to alleviate immunosuppression in the tumor microenvironment, leading to an increased ratio of cytotoxic T cells to regulatory T cells and improved immunotherapy outcomes. pH- and thermo-responsive nanogels loaded with DOX and curcumin have also been designed to enhance treatment outcomes in CRC by sensitizing tumor cells to DOX and reducing drug distribution in healthy tissues [94]. Additionally, nanogels can achieve sustained drug release, improving therapeutic effects while minimizing side effects. A curcumin-loaded nanogel demonstrated enhanced tumor growth suppression compared to free curcumin, highlighting the potential of nanogels in optimizing CRC treatment [95].

#### 3.2.3. Liposomes

On the other hand, liposomes are synthetic vesicles composed of a lipid bilayer that encapsulates aqueous compartments, which have shown promise in CRC therapy (Figure 4B). These spherical nanocarriers, similar to cell membranes, have been FDA-approved for clinical use [96]. Liposomes loaded with tea polyphenols have demonstrated efficacy in treating *Helicobacter pylori* infection, a major contributor to gastric cancer [97]. Furthermore, the encapsulation of polyphenolic compounds, such as resveratrol and EGCG, in liposomes has improved their stability and anticancer performance in prostate and bladder cancer cells [98,99]. Liposomes can also be tailored for drug delivery in the gastrointestinal environment, providing enhanced stability and bioavailability for therapeutic agents like resveratrol and artemisinin [100]. This approach has shown cytotoxic effects on intestinal adenocarcinoma cells, presenting a potential strategy for treating CRC.

#### 3.2.4. Nanoemulsions

Nanoemulsion polyphenol is a specialized structure comprising nanoscale droplets suspended within a continuous phase. This unique system involves the combination of two immiscible phases, typically oil and water, which are held together by an emulsifying agent or surfactant. Within the nanoemulsion, polyphenols, such as quercetin, are loaded into the oil phase (Figure 4B). Notably, the use of quercetin nanoemulsion has shown remarkable efficacy in inhibiting the viability of CRC cells in a dose-dependent manner, surpassing the effectiveness of the drug alone [101]. Furthermore, it has been observed that the nanoemulsion significantly enhances cellular toxicity against CRC cell lines, particularly HT-29 and HCT-116, resulting in more efficient cell eradication compared to the free polyphenol agents [101]. Furthermore, in vivo studies have demonstrated that the administration of quercetin emulsion and nanoemulsion can effectively restore the oxidant-antioxidant balance in mice serum samples and reverse the 5-fluorouracil-induced histological damages in intestinal tissue [102]. These findings highlight the significant potential of quercetin nanoemulsion as a promising therapeutic strategy for CRC treatment.

#### 3.2.5. AuNPs

In the same context, AuNPs have also emerged as promising nanocarriers in the field of CRC treatment due to their advantageous characteristics, including biocompatibility, stability, and the ability to be easily functionalized [103]. These properties make AuNPs an attractive platform for targeted drug delivery [103]. By conjugating polyphenolic compounds onto the surface of AuNPs, a versatile system for delivering therapeutic agents to specific targets is created (Figure 4C) [104]. Numerous studies have demonstrated that polyphenol-coated AuNPs exhibit enhanced cellular uptake and improved bioavailability of therapeutic agents in CRC cells [104]. For instance, the conjugation of EGCG with AuNPs has shown promising anticancer effects in CRC [105]. These effects include induction of cell cycle arrest, promotion of apoptosis, downregulation of NF-κB, and inhibition of tumor growth [105]. The combination of EGCG with AuNPs leads to synergistic therapeutic outcomes, suggesting its potential as an effective strategy for CRC treatment. Moreover, the unique optical properties of AuNPs allow them to serve as photoresponsive agents in photothermal therapy for CRC [103]. By harnessing these properties, AuNPs can selectively destroy tumor cells while sparing healthy tissues.

#### 3.2.6. MSNs

MSNs have gained significant attention as drug carriers due to their porous surface, low toxicity, and high drug-loading capacity [106,107]. Different gatekeepers have been utilized to develop controlled release systems based on MSNs [30,107]. However, challenges such as complex preparation processes and premature drug release still exist. To overcome these challenges, polyphenols have emerged as functional coatings on MSNs. Polyphenol-coated MSNs offer tumor targeting and controlled release properties, making them effective and biocompatible nanocarriers for drug delivery (Figure 4C) [108]. For instance, EGCG-modified MSNs have been developed for drug delivery, where the EGCG coating enhances stability, prevents premature drug release, and provides a site for the immobilization of a DNA aptamer for targeted delivery [109]. The polyphenol coatings are physiologically stable and can be degraded under specific conditions, leading to the release of drugs and subsequent cell apoptosis [109]. Other responsive gatekeepers, such as PDA and magnetic particles, have also been employed on MSNs, further expanding their applications in controlled drug delivery and chemotherapy [110,111].

#### 3.2.7. MOFs

MOFs have gained popularity as hybrid porous materials for drug delivery due to their excellent characteristics such as a porous structure, modifiable components, and satisfactory drug loading capacity (Figure 4C) [112]. However, the clinical application of nanoscale MOFs in cancer treatment faces challenges related to protein binding during circulation and low tumor selectivity. To address these issues, modifications are needed to enhance the bio-stability and tumor targeting of MOFs [113].

Polyphenols, particularly PDA, have been extensively studied as coating materials due to their high affinity to surfaces, photothermal conversion effect, and biosafety [114]. For instance, MIL-100, a pH-sensitive degradable MOF, has been coated with HA-PDA to improve the dispersity, biostability, and tumor-targeting capacity of the NPs [115]. Another study utilized zeolitic imidazolate framework-8 (ZIF-8) as a removable template to construct nanocapsules for efficient drug delivery. The ZIF-8 NPs were decorated with an EGCG-Fe(III) coating, resulting in DOX-encapsulated EGCG-Fe(III) nanocapsules. These nanocapsules could be internalized by cancer cells and release drugs in response to the overproduction of ROS in cancer cells [116].

In Table 1, are presented examples of polyphenol inherent anticancer activity and their advancements in NPs-based drug delivery systems for CRC treatment.

**Table 1:** Other examples of polyphenol-intrinsic anticancer activity and their advancements in nano-based drug delivery systems for CRC.

	**Polyphenols-Intrinsic Anticancer Properties**				
**Polyphenol**	**Source**		**Mechanisms**	**Type of Studies**	**Refs.**
EGCG	Green tea		Modulating gut microbial composition; Down regulation and inhibition of VEGF, EGF, COX-2, p-HER2, ERK, Akt, c-Myc, and Cyclin D1; Gut microbiome modulation;	In vitro and in vivo	[14,15,16,17,18,19,20]
Quercetin	Green tea, onion, etc.		Down regulation of Bcl-2 and triggering apoptosis; Inhibition of Akt pathway; Inhibition of c-Jun N-terminal kinase pathway.	In vitro	[21,22]
Tannic acid	Nutgalls		G0/G1 cell cycle arrest; Inhibiting of TGF-β1/TGF-β1R axis, VEGF/VEGFR axis, JAK/STAT signaling pathway, and CXCL12/CXCR4; Induction of Bak/FADD ratio, p53, p21, p27, p18, Bax, caspases, and endoplasmic reticulum stress.	In vitro	[23]
Resveratrol	Grapes, red wine, and peanuts, etc.		Inhibition of Akt, mTOR, RAS, and ERK; Inducing apoptosis, autophagy, and necroptosis; Cleavage of PARP; Cleavage of caspase-3 and caspase-8.	In vitro	[24,25,26]
	**Nano-Based Drug Delivery Systems of Polyphenols**				
**Polyphenol**	**Nanocarriers/Nanosystem**	**Therapy Strategies**	**Mechanisms**	**Type of Studies**	**Ref.**
EGCG, (+)-catechin hydrate, procyanidin, or ellagic acid	BSA and boronic acid decorated polymer (Figure 2A and Figure 3A)	Targeted gene delivery	Protection of the single-strand nucleic acids from enzymatic degradation; Facilitate the delivery of single-strand oligonucleotides; Enhancement of the efficiency of gene-silencing.	In vitro	[27]
dOEGCG	MMP-2 sensitive NPs (Figure 2C and Figure 3C)	Immunotherapy/PDT	Accumulation at the tumor sites; ROS generation; Apoptosis and necrosis; Blocking the cell surface-expressed PDL1; Sensibility to MMP-2; Accumulated at the lymphatic system; Intratumorally secretion of TNF-α, IFN-γ, IL-1β; Intratumor infiltration and proliferation of CD8+ T cells; Up regulate memory T cells (TCM; CD3+CD8+CD44+CD127+); Inhibited tumor metastasis.	In vitro and in vivo	[28]
Quercetin	Cyclodextrin-based nanoformulation	Chemotherapy/Immunotherapy	Immunogenic cell death induction (ICD); Endoplasmic reticulum stress; Activation of IRE1, ERK and ATF6; Upregulation of p-IRE1; Activation of caspase-3 and caspase-9; CRT, ATP secretion, and HMGB1 release; Autophagy; Upregulation of CD8+ T cells, CD4+ T cells; Activation DCs; Activation of antitumor immunity; Downregulation of Tregs, MDSCs, and M2.	In vitro and in vivo	[29]
Tannic acid	Metal–phenolic network (Figure 2E and Figure 3B)	Chemotherapy/Immunotherapy	Induction of Fenton reaction; ROS generation; Apoptosis induction; CRT, ATP secretion and HMGB1 release; Release/exposure of DAMPs; Maturation of DCs; M1-like repolarization of macrophages; Immunogenic cell death; Increase the proportion of the mature DCs (CD11c+CD80+CD86+) and M1-like macrophages in the primary and distant tumor; Downregulation of Tregs, MDSCs and M2.	In vitro	[30]
Quercetin	Nanoemulsion	Chemo-therapy	Excellent release rate; Inhibition of CRC cell viability; Enhance the cellular toxicity.	In vitro	[101]

### 3.3. Challenges Related to Nano-Based Delivery Systems

The use of various nanocarriers, including micelles, nanogels, liposomes, nanoemulsions, AuNPs, MSNs, and MOFs presents a unique challenge for polyphenol delivery. Indeed, encapsulation and efficient loading of polyphenols within these nanocarriers can be influenced by factors such as polyphenol solubility and compatibility with the carrier system [30]. Achieving controlled release kinetics that match therapeutic needs while preserving polyphenol stability is another hurdle. Biocompatibility and potential toxicity are critical considerations for ensuring the safe use of these nanocarriers in polyphenol delivery [30]. Stability and degradation issues may also arise, impacting the performance and drug release properties of the carriers. Furthermore, scaling up the manufacturing processes while maintaining consistent quality, reproducibility, and control over important parameters poses additional challenges [30]. Overcoming these obstacles through rigorous research and development efforts will advance the field and maximize the potential of nanocarriers for effective polyphenol delivery in biomedical applications.

## 4. Conclusions and Future Prospects

The use of NPs as drug delivery systems in CRC treatment holds tremendous promise. The diverse range of nanocarriers, such as micelles, nanogels, liposomes, and AuNPs, demonstrate their ability to effectively deliver polyphenolic compounds to tumor sites, improving therapeutic outcomes. However, several challenges need to be addressed for the clinical translation of these systems. Stability issues, including oxidation and polymerization, pose a significant hurdle that requires innovative approaches to enhance the stability of polyphenols during fabrication and storage. Moreover, the interactions between polyphenols and biological components should be carefully regulated to avoid non-specific binding and maintain the integrity of the nanocarriers. Further research is also needed to explore the unique interactions offered by different phenolic moieties within polyphenols, allowing for a better understanding of their structure–property relationships and optimization of drug delivery systems. Looking ahead, prospects include the development of natural polyphenolic mixtures-based formulations, cost-effective isolation methods, and the integration of multi-modal imaging and stimuli-responsiveness in nanocarriers. With continued advancements in this field, natural polyphenols have the potential to revolutionize CRC therapy, providing targeted and efficient delivery of therapeutic agents while minimizing side effects and improving patient outcomes.
